# Surveillance of *Escherichia coli* From Frozen Chicken Meat in Fiji: Resistance Characteristics and Public Health Concerns

**DOI:** 10.1155/ijfo/5487064

**Published:** 2025-12-19

**Authors:** Deepika Darshani Lata, Rana Fahmi Sabala, Akira Fukuda, Chie Nakajima, Yasuhiko Suzuki, Masaru Usui, Muhammad Asif Zahoor

**Affiliations:** ^1^ School of Veterinary Medicine, Rakuno Gakuen University, Ebetsu, Hokkaido, Japan, rakuno.ac.jp; ^2^ Department of Food Hygiene, Safety, and Technology, Faculty of Veterinary Medicine, Mansoura University, Mansoura, Egypt, mans.edu.eg; ^3^ Division of Bioresources, Hokkaido University International Institute for Zoonosis Control, Sapporo, Hokkaido, Japan; ^4^ Division of Vaccinology for Clinical Development, Hokkaido University Institute for Vaccine Research and Development, Sapporo, Hokkaido, Japan; ^5^ Division of Research Support, Hokkaido University Institute for Vaccine Research and Development, Sapporo, Hokkaido, Japan

## Abstract

Antimicrobial resistance (AMR) is a growing concern in human and veterinary medicine. Misuse and overuse of antimicrobials in human medicine, veterinary medicine, agriculture, and aquaculture are major drivers of AMR development, with resistant bacteria also being selected in livestock and transmitted through meat. Research on AMR in livestock and animal‐derived foods is lacking in Fiji; thus, the associated risks remain unclear. Chicken is widely consumed in Fiji and is predominantly served frozen. This study is aimed at determining the prevalence and resistance profiles of *Escherichia coli* in frozen chicken meat from Fijian supermarkets. A total of 100 frozen chicken meat samples were purchased from supermarkets and retail outlets in Fiji for this study. *E. coli* was isolated from 72% of the samples. The *E. coli* isolates showed relatively high levels of resistance to ampicillin (36%), tetracycline (24%), and streptomycin (17%). Only one cefotaxime‐resistant isolate was obtained, which was identified as an extended‐spectrum *β*‐lactamase (ESBL)–producing bacterium. This isolate harbored the ESBL‐producing gene *bla*_CTX-M-1_ and was classified as ST2522. One colistin‐resistant isolate was obtained, and its resistance was attributed to a chromosomal mutation in the *pmrB* gene. The high level of intestinal bacterial contamination in frozen chicken meat suggests that improved hygiene management is necessary throughout the production and distribution chains. Furthermore, because resistance to antimicrobials is important in both human and veterinary medicine (cefotaxime‐ and colistin‐resistant *E. coli*), careful monitoring of AMR trends in Fiji is essential. These results suggest that AMR surveillance in meat and livestock is necessary to prevent its spread in Fiji.

## 1. Introduction

The spread of antimicrobial resistance (AMR) poses a significant threat to global health, as it reduces the effectiveness of antimicrobials used to treat infections in humans and animals [1]. AMR bacteria can be transmitted from animals to humans through the consumption of contaminated food [2]. Therefore, effective AMR countermeasures require an understanding of the actual situation and interventions along the food chain.

Chicken is a popular meat product in Fiji [3]. In most supermarkets and retail outlets, chicken meat is predominantly frozen. Fiji has an estimated 3.7 million live chickens, which meet approximately 80% of the domestic demand for chicken products [4]. Chicken meat is commonly contaminated with bacteria, including *Escherichia coli,* during handling, improper dressing, cleaning, and unhygienic practices in meat retailing [5]. Therefore, there are concerns about foodborne illnesses and the spread of AMR in chicken meat in Fiji.

Although some countries have been collecting data on AMR, there is a notable lack of research on its use in livestock production in Fiji [6]. The prevalence of AMR in food animals in Fiji remains unclear, largely because of limited veterinary services and a lack of awareness of AMR among animal health professionals [7, 8]. *E. coli* is commonly used as an indicator for AMR monitoring. It is a commensal microorganism in the gastrointestinal tract of humans and many animals and is widely recognized as a reliable indicator of fecal contamination [9].

This study is aimed at clarifying the prevalence and resistance profiles of *E. coli* in frozen chicken meat in Fiji by isolating and characterizing *E. coli* from domestic and imported frozen chicken meat.

## 2. Material and Methods

### 2.1. Collection of Samples

One hundred frozen chicken meat samples were purchased from retail outlets, butchers, and supermarkets in Fiji between January and March 2024. Samples were collected from seven towns and cities (Nadi, Lautoka, Sigatoka, Suva, Nausori, Labasa, and Savusavu) (Table S1). The samples included whole birds, cut pieces of chicken, minced chicken, thighs, drumsticks, and chicken breasts. Of the 100 samples collected, 80 were from locally produced frozen chicken, and 20 were from imported frozen chicken meat originating from New Zealand. The samples were transported under sterile conditions to the Animal Health Laboratory of the Biosecurity Authority of Fiji in Japan.

### 2.2. Isolation of *E. coli*

After thawing, 25 g of the sample was cut into small pieces using sterile scissors and a scalpel and homogenized with 225 mL of Trypticase Soy Broth (Oxoid, Thermo Fisher Scientific Inc., United States). The mixture was thoroughly homogenized and incubated overnight at 37°C. A loopful of the enriched culture was streaked onto CHROMagar ECC (Kanto Chemical, Tokyo, Japan) and incubated at 37°C for 18–24 h. Three blue colonies, presumptively identified as *E. coli,* were selected and subcultured for further analysis.

### 2.3. Confirmation and Identification of the *E. coli* Isolates

Bacterial identification was performed using matrix‐assisted laser desorption/ionization time‐of‐flight mass spectrometry (MALDI‐TOF MS) with a Bruker MALDI Biotyper system (Bruker Daltonics, Bremen, Germany), following the manufacturer′s instructions and the method described by Croxatto et al. [10].

### 2.4. Antimicrobial Susceptibility Testing

The antimicrobial susceptibility of the confirmed *E. coli* isolates to 11 antimicrobials from eight different classes (ampicillin, cefazolin, cefotaxime, streptomycin, gentamicin, kanamycin, tetracycline, nalidixic acid, ciprofloxacin, colistin, and chloramphenicol; all sourced from Sigma‐Aldrich) was tested. The minimum inhibitory concentrations (MICs) of ampicillin, cefazolin, cefotaxime, streptomycin, gentamicin, kanamycin, tetracycline, nalidixic acid, ciprofloxacin, and chloramphenicol were determined using the agar dilution method for all isolates according to the Clinical and Laboratory Standards Institute (CLSI) guidelines (2023). The MIC of colistin was determined using the microbroth dilution method according to the CLSI guidelines [11]. MIC values and dilution ranges were interpreted according to the CLSI breakpoints (2023) (except for streptomycin, for which the National Antimicrobial Resistance Monitoring System [NARMS] guidelines [2011] were followed). Isolates resistant to three or more antimicrobial classes are considered multidrug‐resistant [12].

### 2.5. Phenotypic and Molecular Detection of ESBL‐Producing *E. coli*

*E. coli* isolates resistant to ampicillin, cefazolin, and cefotaxime were tested for ESBL production using a double‐disc synergy test (DDST) according to the CLSI guidelines [11]. Paired discs of cefpodoxime (10 *μ*g), ceftazidime (30 *μ*g), cefotaxime (30 *μ*g), and aztreonam (30 *μ*g) were positioned at a distance of 20 mm (center to center) from the amoxicillin + clavulanic acid disc (AMC) (20 + 10 *μ*g) (Nissui Pharmaceutical Co. Ltd., Tokyo, Japan). Isolates resistant to cefotaxime and positive for DDST were screened for the presence of *bla*_CTX-M_ using multiplex and singleplex PCR [13, 14]. The PCR products were purified using AMPure XP (Beckman Coulter, United States) and sequenced in both directions using the same primers designed for *bla*_CTX-M_ gene amplification. Nucleotide sequences were determined using the BigDye Terminator v3.1 Cycle Sequencing Kit (Thermo Fisher Scientific Inc.), and DNA alignments and deduced amino acid sequences were examined using the BLAST program (National Center for Biotechnology Information, United States).

### 2.6. Characterization of Colistin‐Resistant *E. coli* Isolates

Isolates resistant to colistin, as determined by the broth microdilution method, were screened for the presence of *mcr* genes (*mcr-1, mcr-2, mcr-3, mcr-4, mcr-5, mcr-6, mcr-7, mcr-8, mcr-9*, and *mcr-10*) using multiplex PCR [15, 16]. Sequence analysis of *pmrB*, a gene associated with colistin resistance, was also performed. The entire *pmrB* gene was amplified for colistin‐resistant isolates using the forward primer 5′‐ACCAACACCCTGGAAGTGCATATCC‐3′ and reverse primer 5′‐TCCTCCAGGTTAACGGAGGAGAGTG‐3′ to amplify a 1304‐bp fragment [15]. The PCR products were subjected to sequence analysis, as described above. Chromosomal mutations associated with colistin resistance were identified using ResFinder (http://genepi.food.dtu.dk/resfinder).

### 2.7. Whole‐Genome Sequence Analysis of ESBL‐Producing *E. coli*

ESBL‐producing *E. coli* isolates were subjected to whole‐genome sequencing analysis. Genomic DNA was extracted using the QIAquick PCR Purification Kit (QIAGEN, Hilden, Germany). The contig was mapped with 150‐bp paired‐end reads obtained using NEBNext Ultra II FS DNA (New England Biolabs, MO, United States) and NovaSeq sequencing platforms (Illumina, San Diego, CA, United States). Short reads were assembled using SPAdes (https://usegalaxy.eu/) with default parameters. Prokka and Staramr were used for sequence annotation and in silico detection of MLST and antibiotic resistance genes (https://usegalaxy.eu/). In addition, mobile genetic elements were detected in the assembled sequence.

### 2.8. Determination Genetic Context of *bla*_CTX-M-1_

The flanking region of the gene was compared with highly similar *bla*_CTX-M-1_ genes available in GenBank (http://www.ncbi.nlm.nih.gov/genebank/). NCBI *E. coli* isolates were recovered from human clinical samples isolated in Denmark (MK181567), Japan (LC567083), and the United Kingdom (CP099077), and from broiler samples studied in France (MG692622) and Switzerland (KJ484637).

### 2.9. Determination of *E. coli* Pathotype by Virulence Genes

The multidrug‐resistant *E. coli* isolates were examined for specific pathotypes based on the virulence genes, including enterohemorrhagic *E. coli* (EHEC) and extraintestinal pathogenic *E. coli* (ExPEC). For EHEC and ExPEC, screening of the following virulence genes was performed using multiplex PCR: *stx1, stx2,* and *eaeA* for EHEC; *cdt, cnf, hlyA, afa, aer, sfa/foc, pap, RPAi, ETTT, IroN, IHA, usp, kps, traT, FyuA, OmpT, ibeA, fimA1*, and *fimH* for ExPEC. PCR was performed as described previously [17]. Based on molecular epidemiological criteria, isolates harboring two or more virulence genes will be classified as potential ExPEC [18].

### 2.10. Statistical Analysis

The variations in the resistance rates of *E. coli* isolates recovered from frozen local and imported chicken meat against the tested antibiotics were assessed using the chi‐square (*χ* ^2^) test and SPSS software v. 23.0 (IBM, Armonk, NY, United States). Statistical significance was set at *p* < 0.05.

## 3. Results

### 3.1. Prevalence of *E. coli* Isolates in the Samples

*E. coli* was isolated from 72% (72/100) of frozen chicken meat samples. The prevalence of *E. coli* in local chicken meat was 65% (52/80), and that in imported chicken meat was 100% (20/20). The contamination rates of the different parts of the collected frozen chicken samples were 51% (18/35) for whole chickens, 89% (8/9) for cut chicken pieces, 100% (3/3) for minced chicken, 75% (9/12) for thighs, 85% (11/13) for drumsticks, and 82% (23/28) for breast meat.

### 3.2. Antimicrobial Susceptibility

Up to three colonies were selected from each sample. Colonies with identical AMR profiles were considered as clones. All colonies isolated from the same sample in this study were clonal, and a total of 72 distinct *E. coli* strains were obtained from the 72 samples from which *E. coli* was isolated.

**Figure 1:**
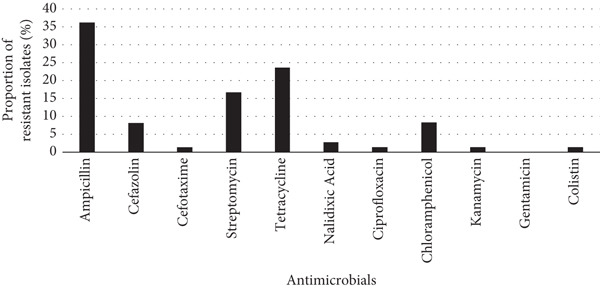
Antimicrobial resistance profiles of *E. coli* (*n* = 72) derived from frozen chicken meat in Fiji: colistin (≥ 4 *μ*g/mL), ampicillin (≥ 32 *μ*g/mL), cefazolin (≥ 8 *μ*g/mL), cefotaxime (≥ 4 *μ*g/mL), streptomycin (≥ 64 *μ*g/mL), kanamycin (≥ 64 *μ*g/mL), gentamicin (≥ 16 *μ*g/mL), tetracycline (≥ 16 *μ*g/mL), nalidixic acid (≥ 32 *μ*g/mL), and ciprofloxacin (≥ 4 *μ*g/mL). Minimum inhibitory concentration (MIC) breakpoints were determined according to the CLSI (2023) guidelines.

Antimicrobial susceptibility testing of the 72 *E. coli* isolates showed that 36%, 24%, and 17% of the isolates were resistant to ampicillin, tetracycline, and streptomycin, respectively (Figure 1). Resistance to cefazolin and chloramphenicol was detected in 8% of the isolates. A lower percentage of isolates (1.4%–2.8%) were resistant to nalidixic acid, ciprofloxacin, kanamycin, cefotaxime, and colistin. All isolates were susceptible to gentamicin. Four isolates (4/72) classified as multidrug‐resistant were resistant to ampicillin, cefazolin, kanamycin, streptomycin, tetracycline, nalidixic acid, ciprofloxacin, and chloramphenicol. All four multidrug‐resistant strains were isolated from local frozen chicken (Table S2). Of these, two were from breast, whereas the remaining two were isolated from drumstick and whole chicken, respectively.

### 3.3. Determination of ESBL Gene

Only one isolate was resistant to cefotaxime (a third‐generation cephalosporin) and was positive for DDST. The isolate produced an ESBL that hydrolyzed third‐generation cephalosporins (such as cefotaxime, ceftazidime, and monobactam aztreonam). However, resistance was inhibited by clavulanic acid, as shown by the inhibition zone around the AMC used in the DDST. The isolate was obtained from a local chicken meat sample. Among the ESBL‐encoding genes screened, the isolate harbored *bla*_CTX-M-1_.

### 3.4. Characterization of Colistin‐Resistant Isolates

Only one isolate was resistant to colistin and tested negative for all screened *mcr* genes. Molecular characterization revealed that colistin resistance in this isolate was due to a chromosomal mutation in the L279M amino acid of the *pmrB* gene, compared with wild‐type *E. coli* strains. This colistin‐resistant isolate was also resistant to ampicillin, cefazolin, streptomycin, and chloramphenicol.

### 3.5. The Whole‐Genome Sequence Analysis of the ESBL‐Producing *E. coli*

In silico analysis based on MLST typing showed that the ST type of the ESBL‐producing *E. coli* isolate, LDDFJ026C, was ST2522. Several antibiotic resistance genes (*bla*_CTX-M-1,_ *sul2, tet* (*A*), and *tet* (*B*)) were detected (Table 1). The plasmid replicon types of this isolate were IncFIA, IncFIA (HI1), IncFIB, IncHI1A, IncHI1B (R27), IncI1‐I, and IncX1.

**Table 1:** Whole‐genome sequence analysis of the ESBL‐producing *E. coli.*

**Isolate**	**Phenotypic resistance profile**	**Resistance genotype**	**MLST**	**Inc type**
LDDFJ026C	Ampicillin, cefazolin, cefotaxime, tetracycline	*bla_CTX-M-1_, sul2, tet (A), tet (B)*	ST2522	IncFIA, IncFIA (HI1), IncFIB (AP001918), IncHI1A, IncHI1B (R27), IncI1‐I (Alpha), IncX1

**Figure 2:**
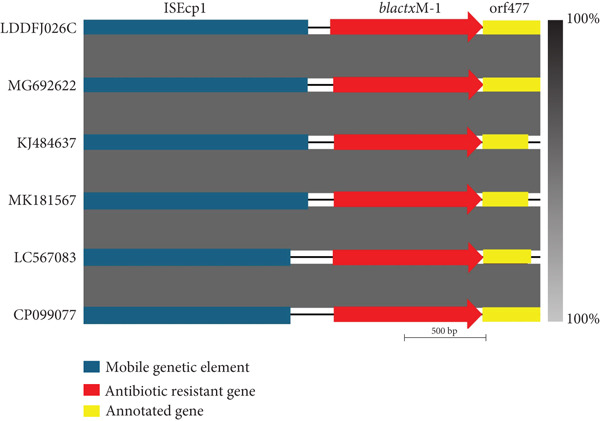
Genomic schematic presentation of the flanking gene regions of the bla_CTX-M-1_ genes in the current study *E. coli* isolate (LDDFJ026C) and the NCBI *E. coli* isolates recovered from a human clinical sample isolated in Denmark (MK181567), Japan (LC567083), and the United Kingdom (CP099077) and from a broiler sample studied in France (MG692622) and in Switzerland (KJ484637). bla_CTX-M-1_: the resistant gene beta‐lactamase CTX‐M‐1; ISEcp1: the IS element IS1380 family transposase ISEcp1; orf477: cupin fold metalloprotein, WbuC family.

The flanking region of *bla*_CTX-M-1_ isolated from strain LDDFJ026C in this study was compared with that of NCBI *E. coli* isolates [19] (Figure 2). All isolates harbored the *bla*_CTX-M-1_ gene, which was encoded by an IncI1 plasmid. The flanking region of *bla*_CTX-M-1_ in *E. coli* 026C isolated in the current study was identical to the flanking region surrounding the *bla*_CTX-M-1_ genes detected in human clinical samples isolated in Denmark (MK181567) carrying ST3 and ST7 [20], in Japan (LC567083) ST3 [21], and in the United Kingdom (CP099077) ST131 and ST10 [22] and from broiler samples studied in France (MG692622) ST3 [19] and in Switzerland (KJ484637) ST3, ST36, and ST1 [23]. Of the six sequences, the gene was flanked by ISEcp1.

### 3.6. Determination of Multidrug‐Resistant *E. coli* Pathotype by Virulence Genes

For the four multidrug‐resistant strains, the presence of virulence genes is shown in Table S2. Based on the virulence gene profiles, two strains were identified as STEC, and all were determined to be ExPEC.

## 4. Discussion

The prevalence of *E. coli* in 100 frozen chicken meat samples from Fiji was 72% (72/100). Similar contamination rates by *E. coli* have been reported in previous studies, at 76% in Korea, 77% in Japan, and 79.68% in the United Arab Emirates [24–26]. High levels of bacterial contamination, especially *E. coli,* in poultry and poultry products are caused by direct or indirect fecal contamination during slaughter, dressing, and processing [27]. Several studies have shown that *E. coli* causes severe foodborne diseases and is transmitted to humans primarily through the consumption of contaminated chicken meat [28, 29]. *Salmonella* spp., *Campylobacter,* and pathogenic *E. coli* cause health problems worldwide, as they are the most important foodborne pathogens of the Enterobacteriaceae family and are transmitted through chicken meat [30, 31]. Therefore, hygienic precautions should be taken in the food processing chain to minimize the contamination of chicken meat by Enterobacteriaceae.

The resistance rates of the 72 *E. coli* strains isolated from frozen chicken meat samples were relatively high for ampicillin, tetracycline, and streptomycin (Figure 1). In the current study, 6% (4/72) of the *E. coli* isolates were classified as multidrug‐resistant. Similarly, a study in New Zealand demonstrated that *E. coli* isolates from poultry were resistant to five antimicrobial classes: aminoglycosides, penicillins, quinolones, tetracyclines, and trimethoprim [32]. These resistant *E. coli* strains have the potential to transfer their resistance genes not only to other *E. coli* strains but also to other bacterial species in the gastrointestinal tract [33]. This highlights the risk of AMR spread within microbial communities, posing a significant public health threat.

The detection of ESBL‐producing *E. coli* in chicken meat in this study was 1.4%. The isolation rate of ESBL‐producing *E. coli* from chicken meat varies depending on the country and the isolation method used [26, 27, 34]; however, the rate observed in this study tended to be lower compared with data reported from other countries. This low prevalence may reflect limited antimicrobial use in Fiji′s poultry industry, where small‐scale production and minimal importation of veterinary antibiotics reduce selective pressure for resistance. A survey of 276 livestock enterprises found that only 56% used antimicrobials, with 22% using only antibiotics [35]. Similarly, a national study in Australia reported no ESBL‐producing *E. coli* among isolates from commercial layer hens [36], suggesting low baseline resistance at the farm level. Continued adherence to prudent antimicrobial use and strengthened biosecurity can help maintain this low prevalence and safeguard both animal and public health.

The cefotaxime‐resistant strain harbored the *bla*_CTX-M-1_ gene. This isolate was recovered from a local frozen chicken meat sample and has been reported for the first time in Fiji. The *bla*_CTX-M_ family of genes, particularly *bla*_CTX-M-1_, is one of the most prevalent in cephalosporin‐resistant bacterial isolates originating from both humans and animals [37]. *bla*_CTX-M-1_ has been recovered from chicken meat samples in many countries, including China [38], United States [39], and Japan [25]. The isolate also harbored tetracycline resistance genes, *tet (A)* and *tet (B).* These genes are found in regions where tetracyclines are used as growth promoters or for prophylactic treatment [40]. In Fiji, beta‐lactams (ampicillin) and tetracyclines are among the most commonly imported veterinary antimicrobials and are frequently used in livestock [6, 41]. The use of antimicrobials in food‐producing animals is a significant driver of the selection and spread of AMR and AMR genes [42–44]. This necessitates continuous AMR surveillance and effective veterinary regulations regarding the use of antimicrobials in food‐producing animals in Fiji.

The ST of the *bla*_CTX-M-1_ gene‐harboring *E. coli* isolate in the current study was ST2522. Previously, *E. coli* isolates of ST2522 have been recovered from fecal samples of sheep in Brazil [45], human clinical samples in the Netherlands [46], and water from vegetable farms in Portugal [47]. Interestingly, there are no previous reports of the isolation of *E. coli* ST2522 from food of animal origin, including chicken meat. These data suggest that ESBL‐producing *E. coli* isolates harboring *bla*_CTX-M-1_ ST2522 are transmitted through the food chain via cross‐contamination. Further investigations are warranted to assess the risks associated with food safety and public health.

Colistin resistance was detected in 1.4% of the *E. coli* isolates. Molecular characterization revealed that no *mcr* genes were present in the isolate, whereas colistin resistance was attributed to a chromosomal mutation showing a substitution of L279M in the *pmrB* gene. Despite the growing awareness of polymyxin resistance and the global prevalence of *mcr-1*, there are few reports on the impact of chromosomal mutations on colistin and polymyxin B resistance in *E. coli* [48]. Furthermore, although amino acid substitutions in MgrB, PmrA/B, and PhoP/Q are common mechanisms of colistin resistance in *K. pneumoniae* in clinical settings, such mutations have rarely been reported in *mcr*‐negative colistin‐resistant *E. coli* isolates [49]. The colistin‐resistant *E. coli* in this study was suggested to be caused by a point mutation in the chromosomal *pmrB* gene; however, further studies are needed to elucidate the mechanisms of chromosomal colistin resistance in *E. coli.*

The isolation of multidrug‐resistant and pathogenic *E. coli* harboring virulence genes indicating the presence of STEC and ExPEC from local chicken meat raises concerns regarding the risk of human infection, and warrants improved hygiene at chicken production and handling facilities.

## 5. Conclusion

This study is the first to report *E. coli* contamination in frozen chicken meat samples from Fiji and evaluate the antimicrobial susceptibility of the recovered isolates. The study revealed a high prevalence of *E. coli* (72%), raising concerns about cross‐contamination of meat, sanitation, hygiene at processing plants, and handling of meat in supermarkets, butchers, and retail outlets. The *E. coli* isolate showed resistance to third‐generation cephalosporins, particularly cefotaxime, despite the extremely small amounts of veterinary antimicrobials imported into the country demonstrating the need to monitor the use of antimicrobials in the veterinary sector. Interestingly, among the recovered *E. coli* isolates, the ESBL‐producing isolate ST2522 harboring *bla*_CTX-M-1_, which was previously recovered from clinical samples, was identified. This indicates that food of animal origin is a potential source of AMR in human infections. Based on this study, regular monitoring and further studies on AMR profiling and prevalence in various food sources of animal origin in Fiji are recommended.

## Conflicts of Interest

The authors declare no conflicts of interest.

## Author Contributions

**Deepika Darshani Lata:** conceptualization, investigation, data analysis, sample curation, resources, and methodology, writing the original draft. **Masaru Usui:** conceptualization, data curation, investigation, project administration, resources, methodology, writing – review and editing. **Rana Fahmi Sabala:** data analysis, curation, writing – review and editing. **Akira Fukuda:** conceptualization, resources, methodology, writing – review and editing. **Chie Nakajima:** supervision. **Yasuhiko Suzuki:** supervision.

## Funding

This work was supported in part by Japan Agency for Medical Research and Development (AMED) under Grant Numbers JP20wm0125008 and JP223fa627005 to Y.S.

## Supporting Information

Additional supporting information can be found online in the Supporting Information section.

## Supporting information


**Supporting Information 1** Table S1: Frozen chicken meat sample collection data in various towns and cities in Fiji.

**Supporting Information 2** Table S2: Pathotype profiling of multidrug‐resistant *E. coli* by detection of virulence genes from frozen chicken meat in Fiji.

## Data Availability

The data that support the findings of this study are openly available in DDBJ at https://www.ddbj.nig.ac.jp/biosample/index‐e.html, Reference Number PRJDB35410.
