# Supplementary material for: Surveillance of Escherichia coli From Frozen Chicken Meat in Fiji: Resistance Characteristics and Public Health Concerns
Source: Int J Food Sci. 2025 Dec 19;2025:5487064. doi: 10.1155/ijfo/5487064 (PMC12715511; doi:10.1155/ijfo/5487064)
Supplement: Supplementary file 1 — Supporting Information 1 Table S1: Frozen chicken meat sample collection data in various towns and cities in Fiji. [file IJFO-2025-5487064-s002.docx]

| Supplementary Table 1. Frozen Chicken meat sample collection data in various towns and cities in Fiji | | |
| --- | --- | --- |
| **Town/ Cities** | **Number of Retail outlets** | **No. of Samples** |
| **Suva** | 10 | 17 |
| **Nausori** | 12 | 43 |
| **Labasa** | 5 | 15 |
| **Savusavu** | 2 | 14 |
| **Nadi** | 3 | 3 |
| **Lautoka** | 3 | 3 |
| **Sigatoka** | 5 | 5 |
